# Faster Is More Different: Mean-Field Dynamics of Innovation Diffusion

**DOI:** 10.1371/journal.pone.0068583

**Published:** 2013-07-19

**Authors:** Seung Ki Baek, Xavier Durang, Mina Kim

**Affiliations:** 1 School of Physics, Korea Institute for Advanced Study, Seoul, Korea; 2 Department of Physics, University of Seoul, Seoul, Korea; University of Nottingham, United Kingdom

## Abstract

Based on a recent model of paradigm shifts by Bornholdt et al., we studied mean-field opinion dynamics in an infinite population where an infinite number of ideas compete simultaneously with their values publicly known. We found that a highly innovative society is not characterized by heavy concentration in highly valued ideas: Rather, ideas are more broadly distributed in a more innovative society with faster progress, provided that the rate of adoption is constant, which suggests a positive correlation between innovation and technological disparity. Furthermore, the distribution is generally skewed in such a way that the fraction of innovators is substantially smaller than has been believed in conventional innovation-diffusion theory based on normality. Thus, the typical adoption pattern is predicted to be asymmetric with slow saturation in the ideal situation, which is compared with empirical data sets.

## Introduction

Pursuing new ideas is a fundamental characteristic of our modern society, where brand-new goods are always ready to push their predecessors off the market. Innovation is one of the most important keywords to understand our society in this sense, as earlier societies were shaped by traditional ideas to be conserved in an unaltered form as much as possible. For this reason, there have been extensive empirical economic and business studies on how innovations get started, diffused and approved in a society, and it is becoming an attractive topic in statistical physics as well [1]–[8]. In a classical work [9] about diffusion of innovations, Rogers claimed that there is a common pattern in innovation dynamics, that people adopting an innovation are normally distributed in time. As a result, the cumulative number of adopters is expected to show an 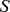-shaped pattern over time, which is described by the error function: It grows slowly at first, expands rapidly at some point, and then slowly saturates to 100%. Deviation from the mean adoption time, 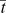, over the entire population defines five adopter categories such as innovators (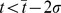, 2.5%), early adopters (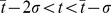, 13.5%), the early majority (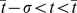, 34%), the late majority (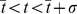, 34%), and laggards (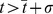, 16%), where 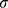 is the standard deviation of adoption time. If normality was true, it might reflect variations in individual innovativeness, which is possibly an aggregate of numerous random events and is normally distributed over the population. However, this is a purely static picture of a non-communicating population and it is an implausible description of an innovative society.

At the same time, Rogers suggested a dynamic origin of this 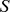-shaped pattern by comparing it to an epidemic process. A relevant description is then more likely to be a logistic function (see, e.g., Refs. [10]–[12]) than the error function. A logistic function is basically written as 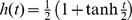, which grows from zero to one as time 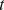 goes from 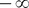 to 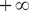. Here, the assumption is that there is a *single* innovation like a disease, diffusing into a passive population. However, the problem with this approach is that ideas are evolving during the course of adoption, and innovation researchers are already well aware that people actively modify an adopted idea whenever it is possible and necessary, which is termed re-invention [13] As a consequence, it is the rule rather than the exception that every modified innovation may well compete with all its predecessors, so the picture becomes more colorful than the dichotomy of a new idea versus an old one. In short, this epidemic description does not capture the genuine dynamic feature of innovations, and even more refined mathematical approaches such as the Bass model do not overcome such limitations [11], [12], [14]. This issue is also deeply related to the pro-innovation bias of diffusion research [9], which means that one tends to overlook such an innovation that dies out by rejection or replaced by a better one. Although there have been statistical-physical approaches to introduce many competing ideas into the dynamics of innovation [3]–[6], they are rather focused on scaling behavior under specific stochastic rules than comparing the findings with empirical observations.

To sum up, analytic concepts are lacking to explain actual patterns of innovation diffusion as a fully dynamic process with a multitude of ideas competing simultaneously. For this reason, we consider simple ideal competition among ideas whose values are so well-defined that everyone can adopt a better idea as soon as she encounters it, without any barriers against the diffusion of innovations. Even if this picture is unrealistic, it is theoretically intriguing, and can serve as a reference point to start with when assessing innovations in practice. In particular, our results suggest that the interplay of adoption and exploration must be considered to achieve a plausible minimalist description, which leads to neither normal nor logistic but slightly skewed behavior as a signature of an ideal innovative society. This simple explanation is in contrast to many variants of the logistic growth model that describe asymmetry in empirical 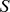-shaped patterns [11], [12]. Moreover, the analysis tells us that the speed of progress in ideas is coupled to how broadly ideas are distributed in the society: a fast innovating society tends to be accompanied by a broad spectrum of ideas, some of which can be far from state-of-the-art. It should be kept in mind that the term ‘ideal’ is absolutely unrelated to any judgments of value concerning the phenomena that we are investigating but only means that we are considering a conceptual construct that can be pursued analytically.

## Methods of Analysis

Following Ref. [7], we assume that every idea is assigned a scalar value 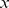 representing its quality. This automatically implies that this quantity is transitive without any cyclic dominance among ideas, and the strict dominance relationship between any pair of distinct ideas prevents people from revisiting old ideas. A difference from Ref. [7] is that 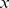 can take any *real* value, not only an integer. Let 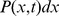 denote the fraction of the population choosing ideas between 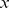 and 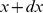 at time 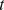. We then call 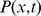 a probability density function (pdf) of idea 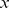. Our population dynamics approach on the mean-field level suggests that the relative growth rate 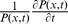 is proportional to the fraction of those with 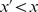 as they are potential adopters of 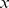. This fraction is, by definition, the cumulative distribution function (cdf) 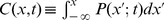 and we thus have
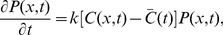
(1)where 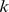 is a positive proportionality constant representing the rate of adoption, which can be set as unity by using a rescaled time 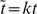, and 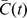 is the average of 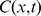 over the population. Note that the total probability is always conserved because 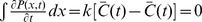 [15]. An alternative way to derive Eq. (1) is to start from a master equation [16]:
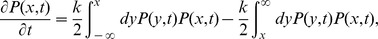
where the first term describes an inflow adopting 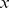 and the second term describes an outflow adopting higher values than 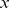. It could also be modified by inserting suitable kernel functions into the integrals.

**Figure 1:**
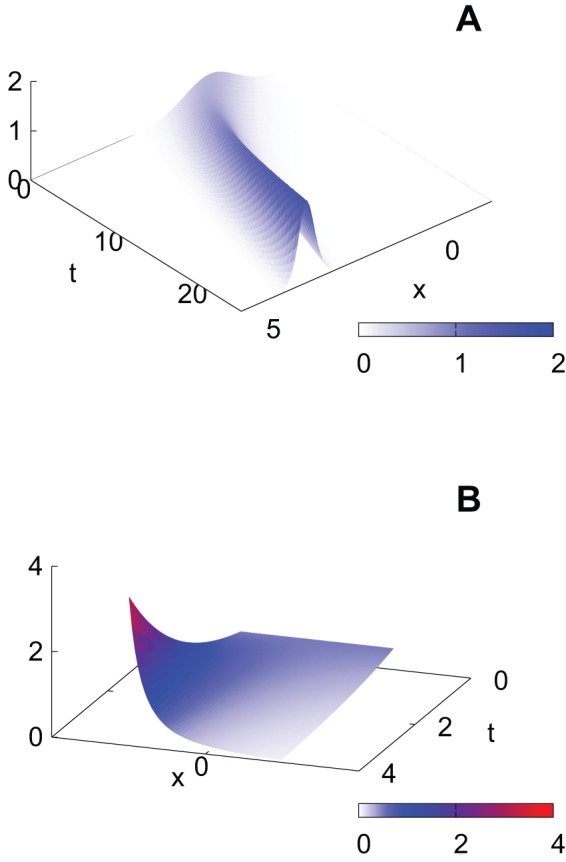
Adoption-only dynamics with different initial conditions. (A) The normal distribution with unit variance [Eq. (7)]. (B) The box distribution defined on 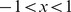 [Eq. (8)].

An integration by parts yields
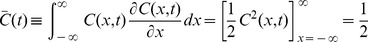
since 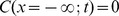 and 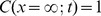. It is convenient to rewrite Eq. (1) only in terms of 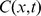:
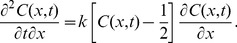
(2)A stationary state with 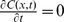 requires 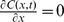 in Eq. (2) since 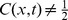 in general due to the boundary condition at 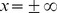. The vanishing derivative with respect to 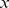 means that 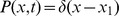 with some constant 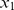, which should be the highest value in the initial pdf with a compact support such that 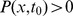 only for 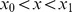 at the initial time 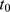. To proceed to the general solution, let us rewrite Eq. (2) as
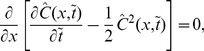
(3)where 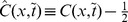 with the rescaled time 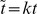. Clearly, Eq. (3) implies that the expression inside the brackets is a function of 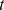 and independent of 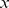. Inserting the boundary condition at 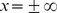, the expression inside the bracket is 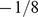 at every 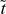. This means that the equation to be solved is the following:
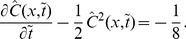
(4)The solution can be found as
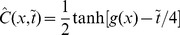
(5)with a certain function 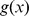. The definition of 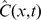 requires 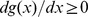 with 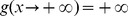 and 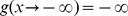. In terms of the pdf, it means that
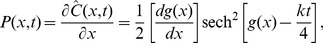
(6)where 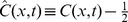 and 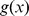 is an arbitrary function satisfying 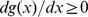 with 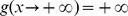 and 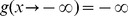. It can be readily checked that it contains the stationary delta function as a special case. If the initial distribution at 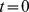 is a normal distribution with unit variance,
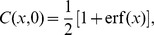
and the time evolution is determined as
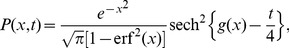
(7)where 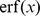 is the error function and 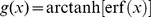. The speed of this wave is 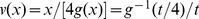, which decreases over time. As the speed decreases, the wave becomes sharper [Fig. 1(a)]. As another example, we take a box distribution defined on the interval between 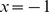 and 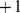 as our initial pdf 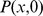. Then we have
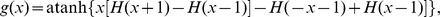
where 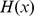 is the Heaviside step function. The solution is given as
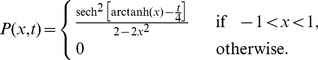
(8)As time goes by, it converges to a delta peak at 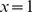 [Fig. 1(b)].

Let us return to the general solution [Eq. (6)]. For any 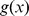 and 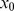, the fraction of the population having passed this innovation level 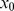, i.e., 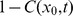, increases as a logistic function of 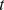. However, it should be noted that our starting point was not meant to be the logistic growth model. The time evolution of 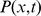 is fully determined once 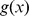 is given by the initial condition, suggesting that innovation history is already determined at the starting point as long as the rate of adoption 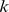 remains unaltered. If the initial condition is nonzero only over a finite range of 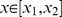, for example, 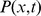 always evolves to a delta function at 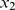. This deficiency makes it difficult to gain insight on the innovation dynamics from the current formulation, revealing its incompleteness.

The reason is that our current formulation does not include any generative mechanism for innovations. Therefore, we add another term to the adoption dynamics considered so far. It could be argued that individual exploration for different ideas can be modeled more or less by a Brownian random walk along the 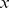-axis:
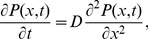
(9)where 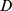 is a measure of exploratory efforts. Because it yields a normal distribution with variance 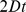, this could be interpreted as invoking the classical idea of normality in the diffusion of innovations, but this normality enters as a consequence of the dynamic exploration process rather than a static trait. It also expresses a conservative viewpoint that an individual alone achieves only small modifications that may even degenerate equally. This is obviously a huge simplification about the human mind, but we shall be content with such a minimalist description at the moment. Adding this exploratory mechanism to the adoption, the resulting equation is written as
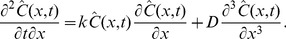
(10)By rescaling 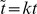 and 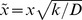, we set both parameters 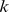 and 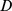 as unity. Notably, Eq. (10) does not have a stationary solution for the following reason: When 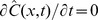, the solution for Eq. (10) is given as Weierstrass' elliptic function, which is even and does not satisfy the boundary condition of 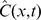 at 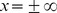. This might look counter-intuitive at first glance as the pdf tends to converge to a single point due to adoption, which could be balanced by exploration. However, a more correct picture is that the pdf converges to a *higher* position than the center, so it gradually moves upward via exploration instead of staying at a fixed position. This notion turns out to be plausible as will be explained shortly below.

If we consider the boundary condition, the actual equation to solve here is given as
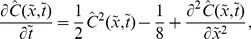
(11)which can be shown identical to Fisher's equation [17] by simply changing the variables. Fisher's equation was originally devised to describe the frequency of a single mutant gene in a one-dimensional population rather than a cdf, and it is interesting that the same equation arises in the context of an infinite series of mutants in an infinite-dimensional (i.e., mean-field) population. This equation has been extensively studied in biology and physics as one of the simplest reaction-diffusion systems [10], [18], [19]. We only mention the basics of the known results about Fisher's equation and those who are interested in comprehensive discussions may refer to Ref. [10] and references therein.

Equation (11) admits traveling wave solutions, and preserves the shapes during propagation. The traveling wave solutions are stable against small perturbations within a finite domain, moving with the waves. Each speed builds up a unique wave shape, and speed 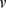 is determined by the tail of the initial cdf in the following manner: If 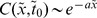 with 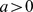 as 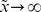 at initial time 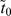, the speed of the wavefront asymptotically converges to 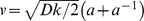 when 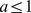, and 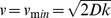 when 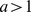. In short, a longer tail leads to a faster propagating wave. Even if an initial pdf has bounded support, i.e., 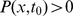 only for 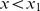, a traveling wave solution will develop with 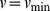 instead of a delta function. The information on the initial condition other than the tail exponent becomes irrelevant in the asymptotic limit due to the random-walk process. There is no traveling wave solution below 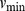, which is consistent with the impossibility of a stationary solution as stated above. Another important feature is that the characteristic width 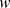 of the wavefront is proportional to 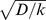 because 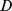 and 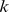 compete to determine width. In contrast, speed is expressed as 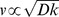 as both the mechanisms of exploration and adoption make positive contributions. As a consequence, the characteristic time for a wavefront to pass through a particular point 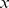 is not sensitive to 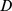 because 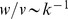.

**Figure 2:**
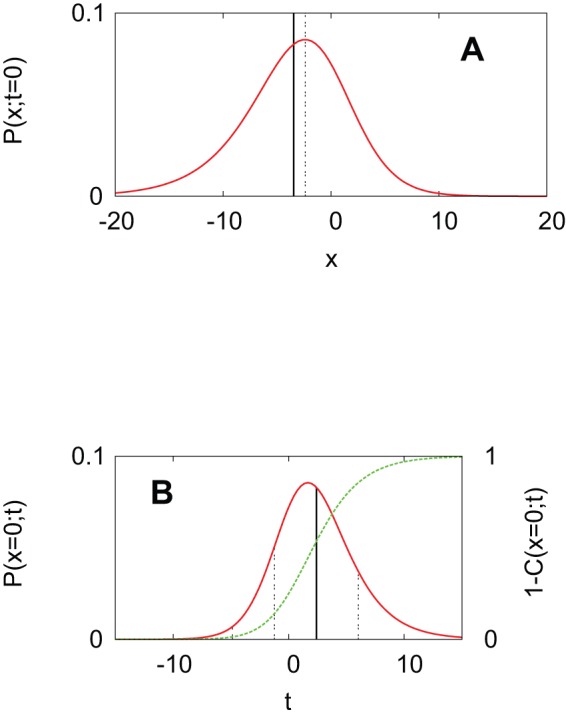
Shape of a traveling wave [Eq. (12)] resulting from Fisher's equation. (A) 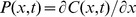 at 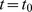 with 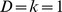. The solid vertical line is the mean, and the dotted vertical line is the mode of the pdf. (B) Temporal pattern of adopting an innovation 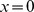 with 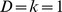 and 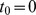. The solid (red) curve 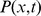 shows how the fraction of the population with 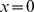 changes over time, whereas the dotted (green) curve 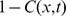 shows the fraction that has adopted 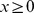 as a function of time. The solid vertical line is the mean adoption time 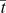, and the dotted vertical lines represent 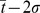, 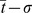, and 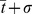, respectively, to distinguish the five adopter categories.

A fully analytic expression for a specific velocity 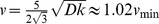 is available as:
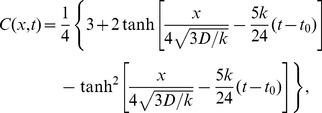
(12)where 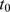 is a reference point in time [20], [21]. As this expression is handy to maintain qualitative features unaltered, we will focus on this solution to observe differences from the normal or logistic descriptions. The numbers presented here should be taken as indicating qualitative features of the solution, and not as universal values for arbitrary 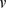. The shape of the wave 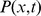 is obtained by differentiating Eq. (12) with respect to 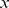, which is shown in Fig. 2(a) at 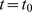. As is clearly shown there, this pdf is not symmetric but skewed negatively, i.e., with a longer tail on the left side. The skewness is quantified from the second and third moments as 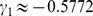. Due to this skewness, while the mean is 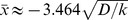, the maximum is located at 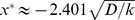. Consequently, the most commonly observed idea tends to lead us to overestimate the population mean. Recall the five categories defined with respect to the *mean* adoption time, which is given by our 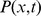 as 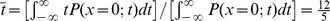, when 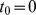 and the idea to adopt has value 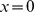 [Fig. 2(b)]. The standard deviation around 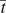 is 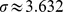, from which we can compute fractions of the five categories as 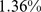 (innovators), 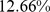 (early adopters), 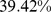 (early majority), 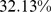 (late majority), and 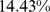 (laggards). Note that the fraction of innovators is only one half of the existing estimate based on the normality. This is due to the inherent skewness of the pdf as a solution for this dynamics. The shape of 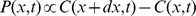 in Fig. 2(b) can also be interpreted as the typical fate of idea 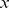, spread by adoption but soon dominated by its descendant 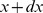.

## Empirical Results

Although Eq. (12) describes only a special case of a specific velocity, we can verify whether it fits to the empirical data set found in Ref. [9]. Recall that a traveling wave with 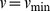 emerges from any initial pdf with a sufficiently short tail, which we presume is close to reality in many cases. Therefore, it would be useful to directly work with this solution, but it is more difficult to handle than the analytic solution Eq. (12) for practical purposes. Fortunately, the analytic solution shows little difference in its shape compared to the solution with 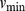. Thus, we work with Eq. (12) to interpret two different data sets : the cumulative number of publications of innovation and the broadband penetration rates in European countries.

### Publications of innovation

**Figure 3:**
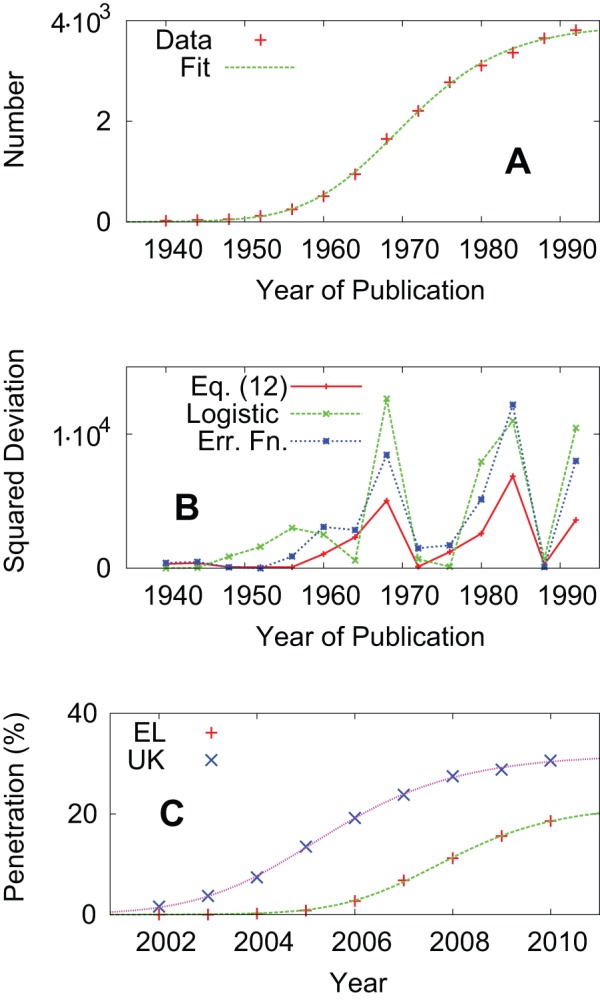
Comparison of Eq.(12) with empirical data. (A) Cumulative number of publications on the diffusion of innovations, excerpted from Ref. [9]. The curve is obtained by fitting a functional form 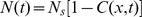 [see Eq. (12)] to the data points where 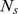 is the saturation number at 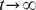. The fitting parameters are 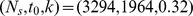. (B) The same data shows larger deviations when fitted with the logistic function 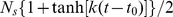 (green) or the error function 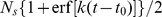 (blue). Their best fitting parameters are 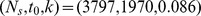 and 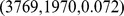, respectively. (C) Broadband penetration rates in Greece and the United Kingdom (UK) from Eurostat [22]. The curves were obtained in the same way as above with Eq. (12), yielding 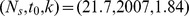 for Greece and 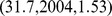 for the UK.

The data set in Fig. 3(a) shows the cumulative numbers of publications on the diffusion of innovations every 4 years from 1940 to 1996. As we approach the late 1990s, the rate of increase decreases, but it is not symmetric with the early take-off around the 1960s. That is, the shape is slightly skewed as our theory suggests [Fig. 2(b)]. The curve in Fig. 3(a) shows our fit of Eq. (12) to the data set by the least-squares method. Although the attempt is quite cavalier, the agreement with the data points is excellent. When compared to fittings with the error function and the logistic function, this functional form actually provides a better explanation, in the sense that the sum of squared deviations becomes one half of each of theirs [Fig. 3(b)]. From this fitting, we can estimate the rate of adoption 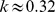. Plugging this value into Eq. (12), we suggest that the relevant time scale of adopting the diffusion concept of innovations amounts to 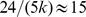 years. One could argue from this excellent fit that the research field is close to the ideal situation that we have considered: researchers are relatively open-minded about new ideas and their communication is not much restricted by geographic factors. Based on this idea, the deviation of empirical adoption patterns from the predicted curve can serve as an indicator to quantify barriers against diffusion of innovations. For example, a classical study of diffusion research on the hybrid corn in Iowa [9] shows a positively skewed pdf contrary to the prediction, which may hint at the strong resistance by the farmers to the new idea at the early stage.

### Broadband penetration in Europe

Our second example in Fig. 3(c) shows broadband penetration rates in European countries, as published by Eurostat [22]. This quantity means the number of high-speed connections (≥144 Kbits/s) per 100 inhabitants. The figure tells us that the broadband penetration in Greece started about 3 years later than that in the UK, and its saturation level in the future will be 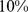 lower than that of the UK. Despite these differences, the relevant time scales of adoption are estimated to be about 3 years for both countries.

**Figure 4:**
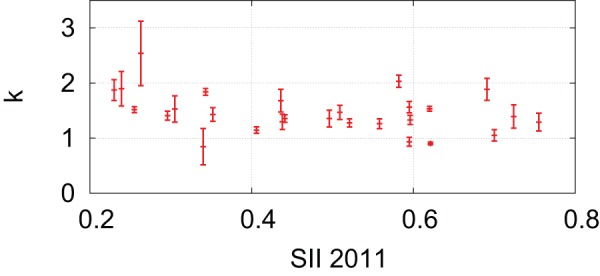
Summary innovation index (SII) versus the rates of adoption in the European Union (EU) member countries.

In fact, the rates of adoption, evaluated from the broadband penetration rates, do not change much across European countries. Table 1 shows the least-square fitting results of Eq. (12) to the broadband penetration rates from 2002 to 2010 in EU member countries [22]. Note that the values in column 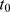 are relative to 2002. In Fig. 4, we plot the resulting 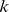 values in Table 1. The horizontal axis represents the summary innovation index (SII), which has been developed to assess aggregate national innovation performance of the EU member countries [23]. It is a composite index showing how many relevant indicators such as education, employment and R&D are above or below EU averages. Figure 4 suggests that the differences in innovativeness measured by the SII cannot be explained by the differences in the rates of adoption. Therefore, if we use the SII as a proxy variable for measuring speed 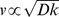, the differences in the SII should be explained by variations in the measure 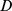 of exploration activity.

**Table 1:** Fitting results of Eq. (12) to the broadband penetration rates from 2002 to 2010 in EU member countries.

Country	*N_s_*	*t*_0_	*k*	Country	*N_s_*	*t*_0_	*k*
BE	33.5±0.4	0.49±0.04	0.90±0.02	LU	34.8±0.9	2.58±0.08	1.56±0.10
BG	15.5±1.0	4.43±0.11	1.90±0.31	HU	22.0±1.0	3.36±0.10	1.43±0.12
CZ	21.4±1.1	3.37±0.13	1.68±0.21	MT	43.4±16.9	3.74±1.15	0.85±0.33
DK	40.7±2.0	0.99±0.21	1.39±0.21	NL	40.5±0.9	1.06±0.08	1.33±0.08
DE	38.5±2.5	2.67±0.18	1.05±0.10	AT	26.9±1.2	1.11±0.14	0.93±0.08
EE	28.5±1.2	1.99±0.13	1.36±0.15	PL	18.5±0.8	4.31±0.08	1.41±0.08
IE	23.7±0.5	3.38±0.05	2.03±0.11	PT	19.8±0.9	1.48±0.15	1.30±0.14
EL	21.7±0.5	4.75±0.04	1.84±0.06	RO	14.0±0.7	4.25±0.19	2.54±0.58
ES	24.9±0.6	1.84±0.08	1.15±0.06	SI	26.8±0.8	2.82±0.07	1.28±0.07
FR	33.9±1.1	2.10±0.10	1.26±0.09	SK	19.1±2.0	4.39±0.20	1.53±0.24
IT	22.3±0.5	1.93±0.07	1.36±0.07	FI	31.1±0.9	1.36±0.13	1.89±0.20
CY	28.0±1.6	4.12±0.11	1.47±0.13	SE	35.4±1.7	1.26±0.17	1.29±0.16
LV	20.1±0.8	3.47±0.10	1.87±0.19	UK	31.7±0.3	2.05±0.04	1.53±0.04
LT	21.5±0.4	3.12±0.04	1.52±0.05				

If 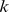 is uniform, our model predicts that more diverse values of 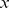 will be observed in a society where innovation occurs faster because both 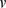 and 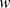 scale as 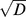. The abundance of laggards with low 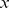 results from the fast innovation but also fuels it as market potential, and both effects are incorporated in the solution.

## Discussion and Summary

In summary, we have studied an ideal innovative society where a better idea has a better chance to diffuse into the population. Our model is characterized by competition among an infinite number of ideas. In the presence of an adoption mechanism only, we are able to find the full solution exhibiting logistic behavior, but it is a purely deterministic view leaving the concept of innovation obscure. By adding another term for exploratory behavior, which connects to the classical idea of normality, we have found traveling wave solutions as described by Fisher's equation, whose velocity is proportional to the square root of exploration activity 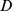 times the rate of adoption 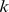. At the same time, its width is proportional to 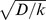 due to the competition of adoption and exploration. Incorporating both the normal and logistic features, the shape of the solution is neither normal nor logistic but negatively skewed, leading to a discrepancy between the mean and the mode as well as a significantly smaller size estimate of innovators compared to that of the conventional theory. It is compared with the asymmetry in empirical adoption patterns and proposed as a reference point to assess the effectiveness in diffusion of innovations. Furthermore, as the rates of adoption do not vary much across countries, we predict a tendency for the width of a distribution to be positively correlated with the overall speed of innovations.
